# Knee joint line related to bony landmarks of the knee: a radiologic study in a Thai population

**DOI:** 10.1186/s43019-022-00135-5

**Published:** 2022-02-15

**Authors:** S. Tantavisut, C. Amarase, S. Ngarmukos, C. Tanavalee, A. Tanavalee

**Affiliations:** 1 Department of Orthopaedic, Chulalongkorn University, Bangkok, Thailand; 2 Hip Fracture Research Unit, Chulalongkorn University, 1873 Rama 4 Road, Lumpini, Pathumwan, Bangkok, 10330 Thailand

**Keywords:** Knee arthroplasty, Revision, Joint line restoration, Bony landmarks

## Abstract

**Background:**

During revision total knee arthroplasty (TKA), knee joint line restoration may be difficult due to bone loss or structural changes. Although bony landmarks are consistent and can be used as references, there are limited data in Asian patients. We studied the knee joint line related to bony landmarks of the knee in a Thai population.

**Materials and methods:**

Magnetic resonance imaging (MRI) of 140 healthy knees of Thai patients (70 males, 70 females) were investigated. In all knees, a perpendicular line from knee joint line to the medial epicondyle (distance *A*) and the lateral epicondyle (distance *B*) in the coronal plane were measured. In the sagittal plane, a perpendicular line from the knee joint line to the fibular head (distance *C*), the tibial tubercle (distance *D*), and the inferior patellar pole (distance *E*) were measured. The femoral transepicondylar width (FW) was measured along the transepicondylar axis. The ratios of distances *A*, *B*, *C*, *D*, and *E* related to FW were evaluated (epicondylar ratio).

**Results:**

The mean and standard deviation (SD) of distances *A*, *B*, *C*, *D*, *E*, and FW were 27.1 ± 2.7 mm, 21.7 ± 2.5 mm, 12.6 ± 3 mm, 21.3 ± 3.6 mm, 7.6 ± 4.8 mm, and 76.7 ± 3.99, respectively. There was wide variation of measured values, with statistically significant differences between genders in distances *A*, *B*, *C*, and FW. The mean and SD of epicondylar ratios *A*/FW, *B*/FW, *C*/FW, *D*/FW, and *E*/FW were 0.35 ± 0.02, 0.29 ± 0.02, 0.16 ± 0.05, 0.28 ± 0.04, and 0.09 ± 0.04, respectively. All epicondylar ratios demonstrated less variation than all measured distances, with statistical differences between genders in the *A*/FW and *D*/FW ratios. However, the *B*/FW ratio had the highest consistent mean value. In addition, it had narrower SD than the rest (0.29 ± 0.02; range, 0.22–0.33).

**Conclusions:**

In Thai knees, the measured distances from bony landmarks to the knee joint line had higher variation than the epicondylar ratio. Among all studied epicondylar ratios, the ratio between lateral epicondyle to joint line distance (distance *B*)/FW demonstrated the narrowest range of mean and SD values; therefore, this could be the most reliable landmark for intraoperative knee joint line verification by multiplying the FW of the patient by 0.29 to get distance *B* in that patient.

## Introduction

Knee osteoarthritis is a common disease in the elderly, for which a total knee arthroplasty (TKA) is an effective treatment option for the late stage. To gain a satisfactory outcome with implant longevity after TKA, several factors must be addressed. Intraoperatively, restoration of the knee joint line is one of the important factors for successful TKA [1, 2]. A change of over 4 mm in the joint level is related to unsatisfactory clinical outcomes, chronic pain, alteration of tibiofemoral joint kinematics, knee joint laxity, and increased wear rate of TKA prosthesis [3–5].

Difficulty in joint line restoration frequently occurs in revision TKA, related to bone loss and osteolysis. The soft tissue landmarks for the knee joint line, such as the meniscal rim, is reported to be unreliable and difficult to reference in actual surgical settings [6], while bony landmarks are reportedly reliable and widely used in revision TKA [7–9]. There are several studies regarding the application of bony landmarks for joint line restoration in difficult primary or revision TKA by researchers worldwide [8–12]. According to gender, ethnicity, and patient’s stature, the reported distances from bony landmarks to knee joint line have high variation [13]. Some authors have suggested that the ratio of the distance from bony landmarks to the joint line and the femoral transepicondylar width (FW), also called the “epicondylar ratio,” is more reliable than the distances from landmarks to joint line [8, 9, 14]; however, studies based on specific ethnic populations have found variations in the proposed ratios [15].

As there are limited studies regarding knee joint line landmarks in Asian patients, we evaluated the relationship of the distances from bony landmarks around the knee to the joint line, as well as the epicondylar ratios between these distances and FW in normal knees of the Thai population.

## Materials and methods

This study was approved by the ethical committee in our institute. We included magnetic resonance imaging (MRI) data of 140 knees in healthy Thai volunteers who had normal lower limb alignment and range of motion. There were 70 males and 70 females, with ages ranging from 18 to 60 years old. In all knees, the magnetic imaging scan covered 15 cm above and below femorotibial articulation with 3 mm slice thickness using a constant magnetic field of 1.5 Tesla with extremity coil (Siemens, Avanto, Germany). The MRI data were precalibrated using a cadaveric specimen to provide zero magnification. All knees were set at full extension during MRI scanning.

**Fig. 1:**
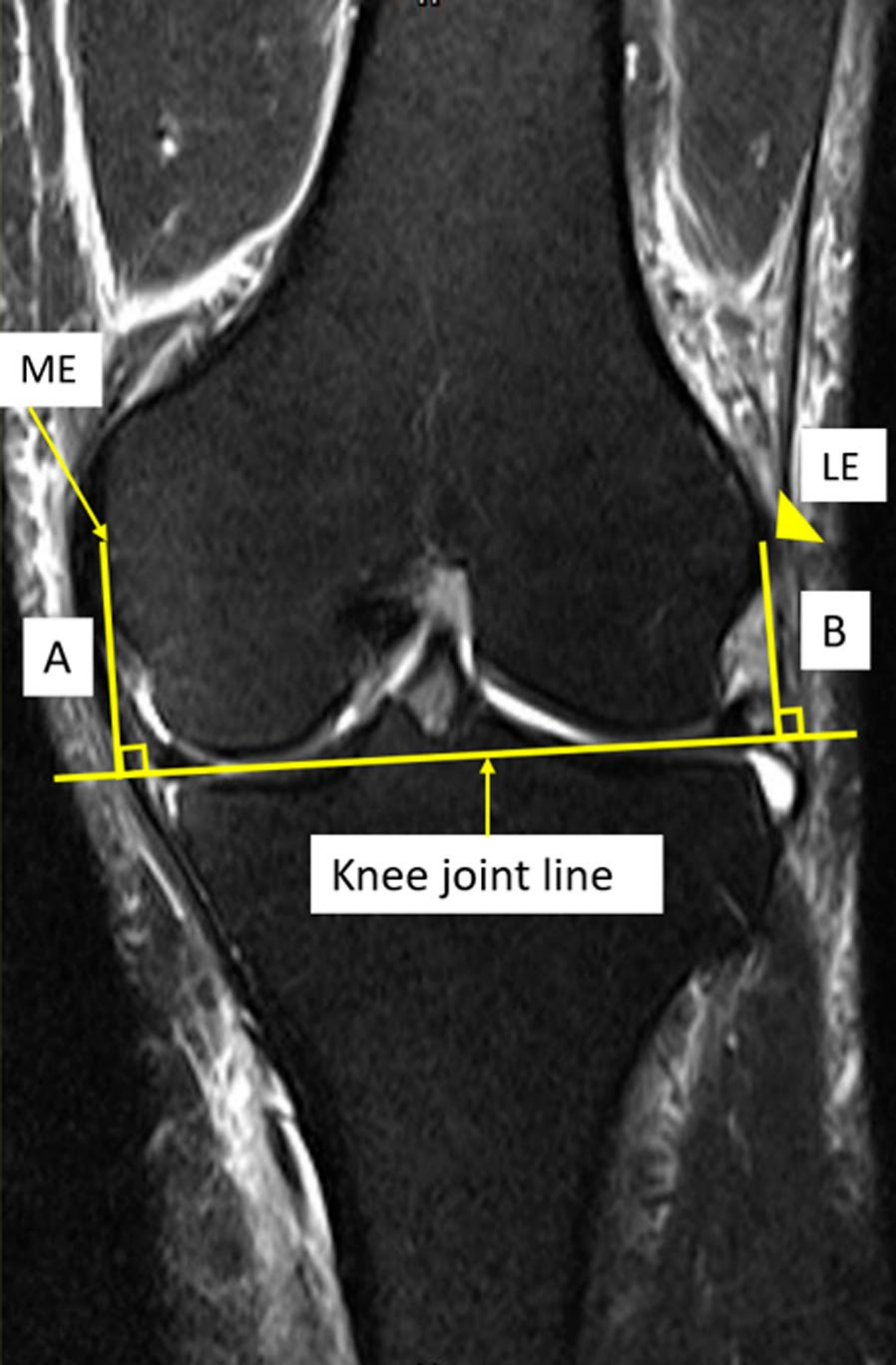
Demonstration of the measurement technique in coronal view of MRI. The knee joint line connects the most distal point of medial femoral condyle and lateral femoral condyle. Line *A* is the distance from the most prominent point of medial femoral epicondyle (ME, arrow) perpendicular to the knee joint line. Line *B* is the distance from the most prominent point of lateral femoral epicondyle (LE, arrowhead) perpendicular to the knee joint line

**Fig. 2:**
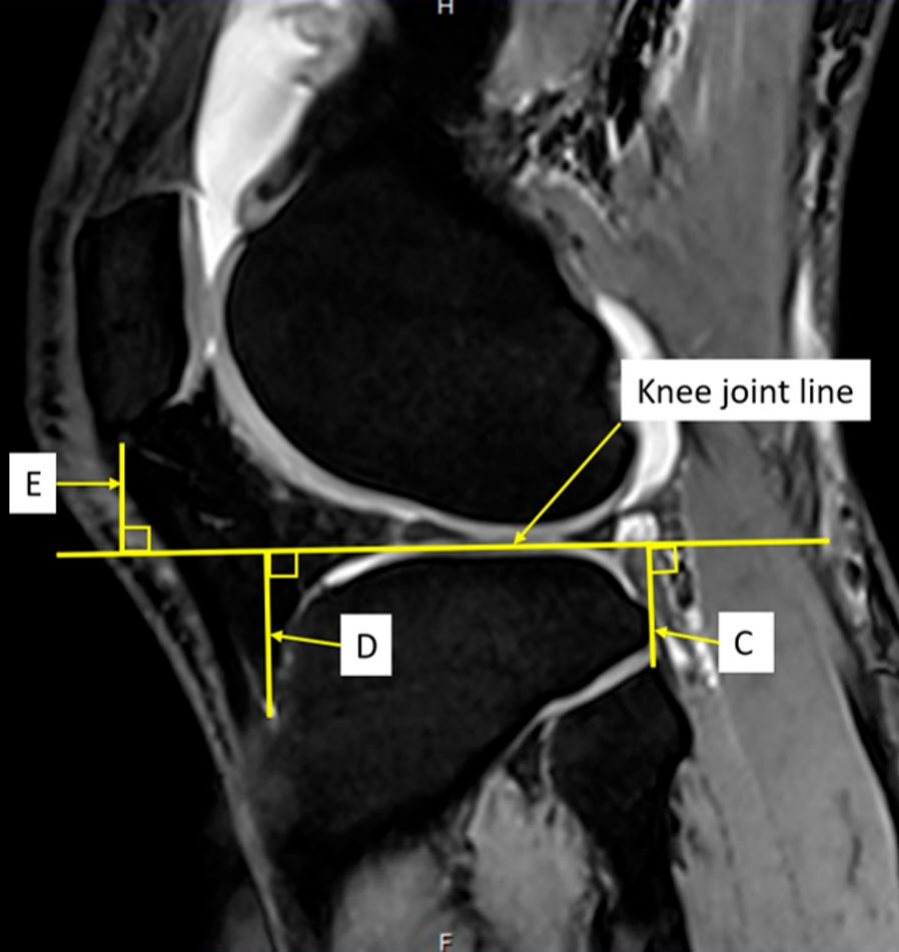
Demonstration of the measurement technique in sagittal view of MRI. The knee joint line was identified by the line between the highest points of cartilage of the anterior and the posterior tibial plateau. Line *C* is the distance from the highest point of fibular head perpendicular to the knee joint line. Line *D* is the distance from tibial tubercle to the knee joint line. It is measured from the most proximal point where the patella tendon is inserted into the tibial tubercle to the knee joint line. Line *E* is the distance from the most inferior point of inferior pole patella perpendicular to the knee joint line

**Fig. 3:**
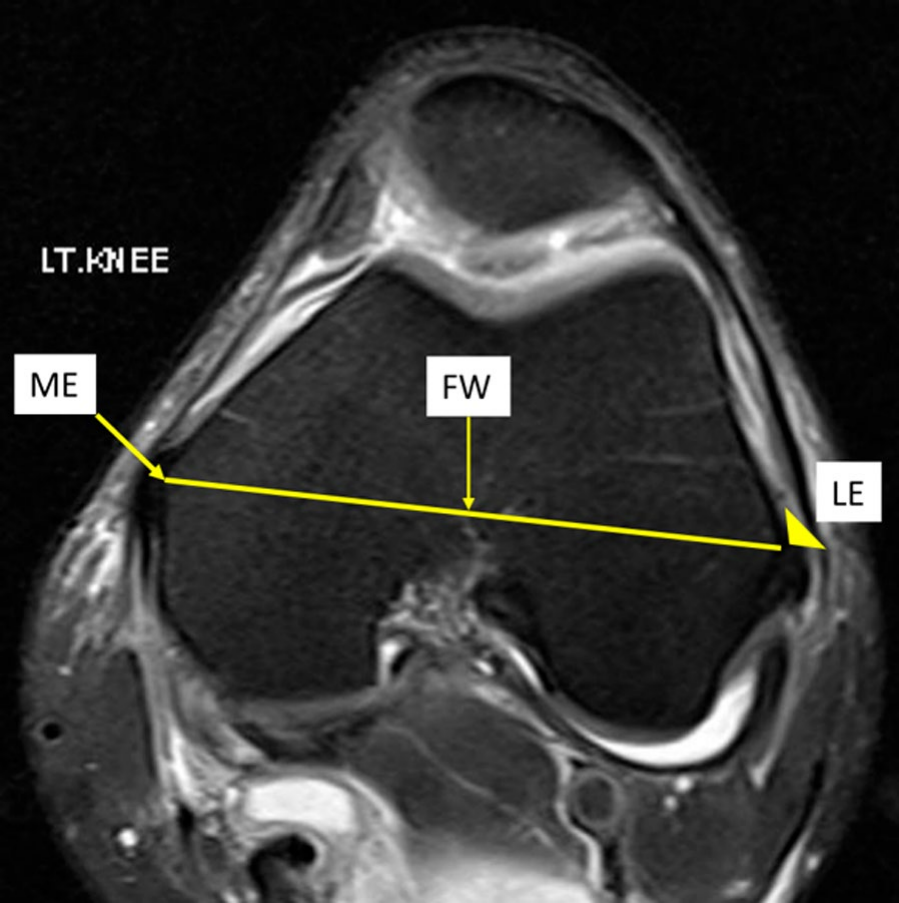
Demonstration of the measurement technique in axial view of MRI. The femoral transepicondylar width (FW) is the distance from the most prominent point of medial femoral epicondyle (ME, arrow) to the most prominent point of the lateral femoral epicondyle (LE, arrowhead)

In coronal view, the knee joint line was identified by the line between the lowest points of cartilage of the medial and the lateral femoral condyles (Fig. 1). In sagittal view, the knee joint line was identified by the line between the highest points of cartilage of the anterior and the posterior tibial plateau (Fig. 2). The perpendicular lines from the knee joint line in coronal and sagittal planes were drawn to several bony landmarks around the knee, including the most prominent point of medial epicondyle (line *A*, Fig. 1), the most prominent point of lateral epicondyle (line *B*, Fig. 1), the highest point of fibular head (line *C*, Fig. 2), the tibial tubercle (line *D*, Fig. 2), and the lowest point of inferior patellar pole (line *E*, Fig. 2). Line *D* was measured from the most proximal point where the patella tendon is inserted into the tibial tubercle to the knee joint line in sagittal view of MRI (Fig. 2). The distances of line *A*, *B*, *C*, *D*, and *E* were measured and defined as distance *A*, *B*, *C*, *D*, and *E*, respectively. The line from the most prominent point of medial and lateral epicondyle was drawn and defined as the femoral width (FW), as shown in Fig. 3. The epicondylar ratios, which are ratios of distance *A*, *B*, *C*, *D*, and *E* related to FW were evaluated. All measurements were performed by two experienced hip and knee reconstruction specialists.

### Statistical analysis

All data were analyzed using IBM SPSS statistics for Windows version 25.0 (IBM Corp, Armonk, NY, USA). Qualitative data were reported as frequency and percentage. The quantitative data were reported as mean ± SD. The differences among the whole group, male gender, and female gender were analyzed with a one-way ANOVA test. The Pearson and Spearman’s coefficients were used to evaluate inter- and intraobserver reliability.

## Results

**Fig. 4:**
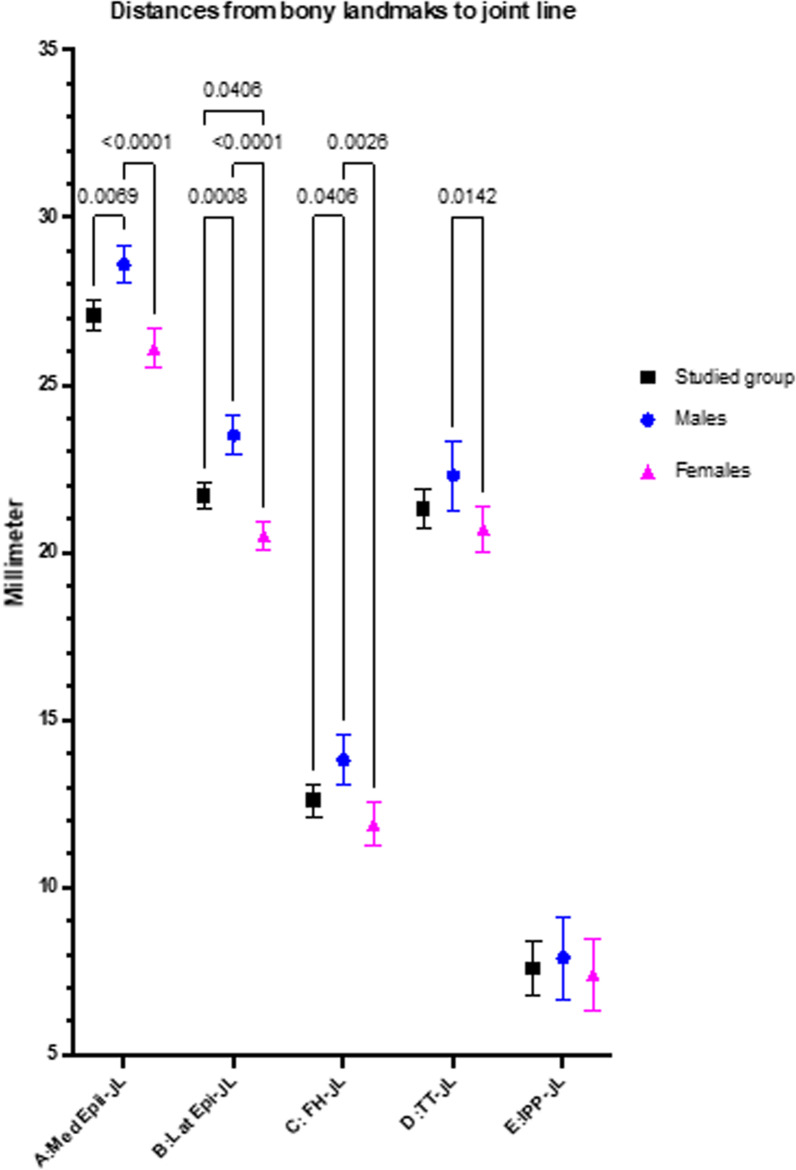
Graph comparing the mean and standard deviation among investigated distances to the knee joint line. There are significant variations between genders in distances *A*, *B*, *C*, and *D*

**Fig. 5:**
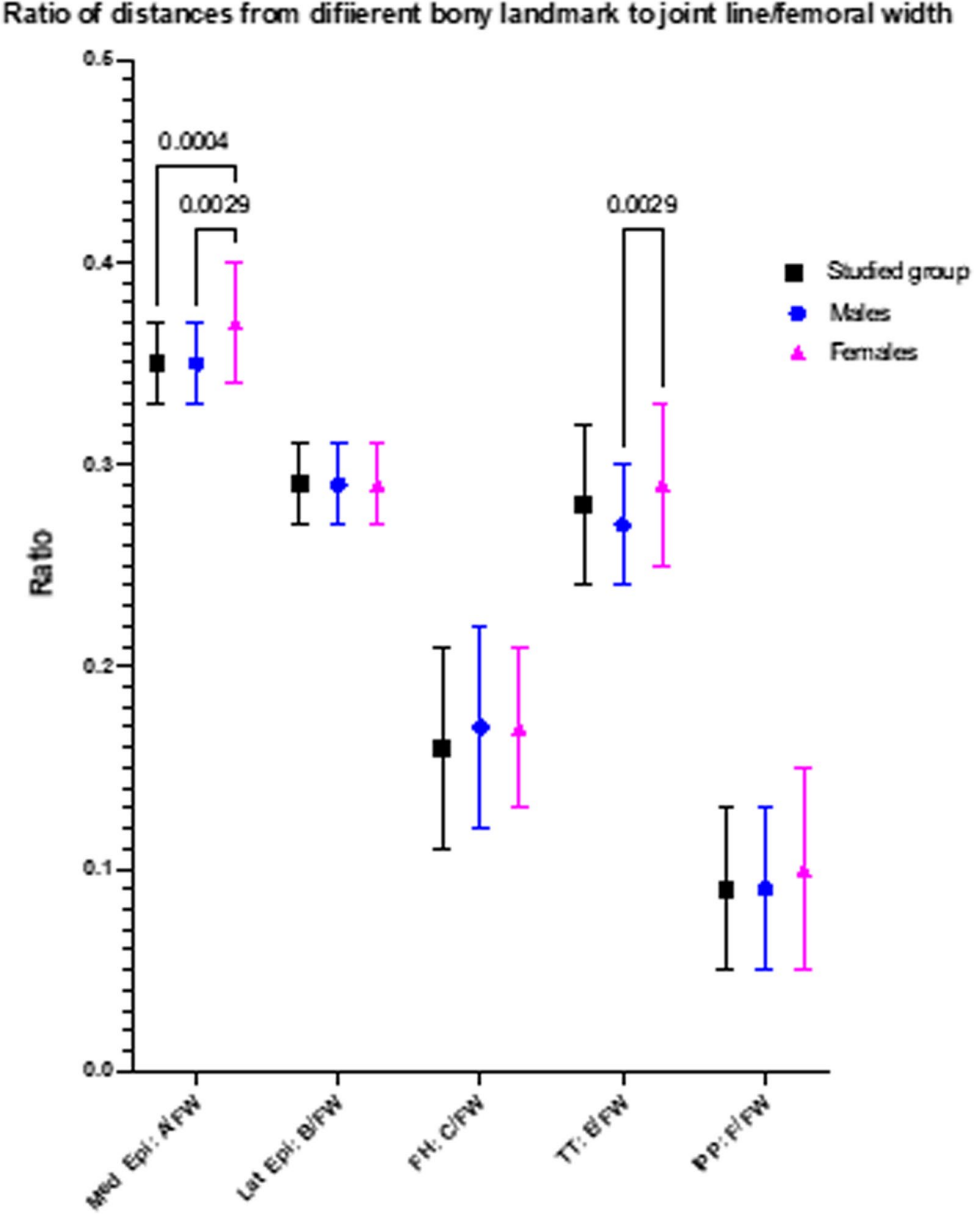
Graph comparing the mean and standard deviation among ratios between investigated distances and the femoral width. The ratio of distance *B*/FW provides a similar mean, with narrower standard deviation than the others

Among 140 knee MRI images, the average age of the studied group was 47.1 ± 8.7 years, with no differences between genders. Demographic data of the participants are presented in Table 1. The mean and standard deviation (SD) of distance *A*, *B*, *C*, *D*, *E*, and FW were 27.1 ± 2.7 mm, 21.7 ± 2.5 mm, 12.6 ± 3 mm, 21.3 ± 3.6 mm, 7.6 ± 4.8 mm, and 76.7 ± 3.99, respectively. There was wide variation in the measured values, with statistically significant differences between genders in distances *A*, *B*, *C*, and FW (Table 2, Fig. 4). The mean and SD of epicondylar ratios *A*/FW, *B*/FW, *C*/FW, *D*/FW, and *E*/FW were 0.35 ± 0.02, 0.29 ± 0.02, 0.16 ± 0.05, 0.28 ± 0.04, and 0.09 ± 0.04, respectively (Table 3). All epicondylar ratios demonstrated less variation than all measured distances, with statistical differences between genders in *A*/FW and *D*/FW ratios. However, among the five epicondylar ratios, the *B*/FW ratio had the highest consistent mean value. In addition, it had a narrower SD than the others (0.29 ± 0.02; range, 0.22–0.33). (Fig. 5). The mean interobserver coefficient was 87% (within 1 mm difference) and the mean intraobserver coefficient was 93% (within 1 mm difference).

**Table 1:** Demographic data

Parameters	Studied group	Male	Female	*p*-Value
Number	140	70	70	1.0
Age (years) (mean ± SD)	47.1 ± 8.7	46.5 ± 8.8	48.1 ± 8.5	0.55
BMI (kg/m^2^) (mean ± SD)	25.5 ± 6.2	25.3 ± 6.5	26.2 ± 8.5	0.78
Side				
Right	70	36	33	0.84
Left	70	34	37	0.76

**Table 2:** Measurement results

Parameters	Total participants	Males	Females	Males versus females *p*-Value
Distance *A*	27.1 ± 2.7 (19.3–34)	28.6 ± 2.4 (22.8–34)	26.1 ± 2.4 (19.3–33.9)	< 0.0001
Distance *B*	21.7 ± 2.5 (16.1–29.2)	23.5 ± 2.4 (17.7–29.2)	20.5 ± 1.8 (16.1–26)	< 0.0001
Distance *C*	12.6 ± 3 (4.5–21.6)	13.8 ± 3.1 (4.5–21.6)	11.9 ± 2.7 (4.5–18)	0.0026
Distance *D*	21.3 ± 3.6 (3–30.7)	22.3 ± 4.3 (3–30.7)	20.7 ± 2.8 (10.8–27.6)	0.142
Distance *E*	7.6 ± 4.8 (−3 to 19.9)	7.9 ± 5.2 (−3 to 18.2)	7.4 ± 4.5 (−2 to 19.9)	0.50
FW	76.7 ± 3.99 (57.9–93.8)	81.44 ± 4.12 (72.8–93.8)	70.7 ± 3.64 (57.9–85.4)	< 0.001

Mean ± SD (range)

All measurements were performed in millimeter

**Table 3:** Epicondylar ratio

Parameters	Total participants	Males	Females	Males versus females *p*-Value
*A*/FW	0.35 ± 0.02 (0.29–0.44)	0.35 ± 0.02 (0.29–0.43)	0.37 ± 0.03 (0.32–0.44)	0.0029
*B*/FW	0.29 ± 0.02 (0.22–0.33)	0.29 ± 0.02 (0.23–0.33)	0.29 ± 0.02 (0.22–0.32)	0.50
*C*/FW	0.16 ± 0.05 (0.05–0.30)	0.17 ± 0.05 (0.05–0.30)	0.17 ± 0.04 (0.09–0.29)	0.45
*D*/FW	0.28 ± 0.04 (0.04–0.37)	0.27 ± 0.03 (0.04–0.37)	0.29 ± 0.04 (0.13–0.36)	0.0029
*E*/FW	0.09 ± 0.04 (0.05–0.27)	0.09 ± 0.04 (0.05–0.26)	0.10 ± 0.05 (0.07–0.27)	0.27

Mean ± SD (range)

## Discussion

The present MRI study investigated distances between the bony landmarks and the knee joint line, including the medial epicondyle, the lateral epicondyle, the fibular head, the tibial tubercle, and the inferior patellar pole. There was wide standard deviation (SD) of all measured landmarks–joint line distances, which might not be suitable as the reference for knee joint line identification in difficult or revision TKA. In contrast, the ratios of investigated distances related to the FW (epicondylar ratio) had less variation in the mean and SD than those of distance measurements. Among all studied epicondylar ratios, *B*/FW demonstrated the narrowest SD range, which could be useful for intraoperative knee joint line verification by multiplying the FW of the patient by 0.29 (Table 3) to get the absolute value of distance *B* in that particular patient.

One of the most important and challenging tasks while performing TKA is to restore the natural joint line level [1, 2]. In primary TKA, the chance of success is high because of the availability of natural bone allowing matched resection and a guide for the joint line level. It is a much more difficult and unreliable task to achieve during revision TKA due to the lack of normal anatomy and distorted anatomy from previous surgery or bone loss. Porteous et al. [16] studied revision TKA and found that if the surgeon can restore the joint line to within 5 mm of the preoperation level, this would result in a significantly better outcome. This is well supported by several studies that suggest that patients with a change in joint line of more than 5–8 mm had worse outcomes, more pain, less motion arc, increased knee instability, and negative effects to the knee extensor mechanism [3–5].

There are several methods that can help the surgeon find the proper joint line in revision TKA. The primary implant can be used as a guide for joint line level before being removed. However, this method can only be utilized if the primary implant has an accurate joint line level [17]. Surgeons can use contralateral knee radiographs as a template and guide for joint level if the radiographs are available and the contralateral knee has no anatomical distortion. In general, the most widely used method to guide the accurate joint line level is using intraoperative landmarks, which can be divided into soft tissue and bony landmarks. Generally, the soft tissue landmark is difficult to locate exactly, and most likely distorted from prior surgery [6]. Bony landmarks are more reliable and available to use even in revision TKA or severe bone loss situations [7–9]. The surgeon can locate bony landmarks intraoperatively then use it as a reference to guide for the correct joint line level [8].

The medial epicondyle, lateral epicondyle, tibial tubercle, fibular head, and inferior pole of the patella are landmarks that commonly used in a clinical setting. Maderbacher et al. [18] investigated the distances from several bony landmarks to the knee joint line and found that the distances from medial epicondyle, lateral epicondyle, and fibular head to the knee joint line were 33.9, 33.4, and 12.2 mm, respectively. Jawhar et al. [19] reported that the distance from fibular head to the knee joint line was 10 mm. Mason et al. [20] suggested that the knee joint line level can be found at the level of inferior pole patellar or 2 cm above the fibular head on an extended knee. For medial epicondyle evaluation, the current study chose to evaluate the most prominent point of medial epicondyle rather than the medial epicondyle sulcus as it is more accurate and easier to locate in a surgical setting [21]. Several studies were identified that evaluated these study parameters but patients of different ethnicity [7, 8] (Table 4). The distances from landmarks in this study are comparable to most of these results, except distances *C* and *E*. The differences in distances *C* and *E* between this study and other studies could possibly be explained by the evidence that both landmarks had high variability. Several previous studies have reported that the fibular head and lower pole patellar are not a good reference to locate the knee joint line due to the variability in their position [8, 9]. In addition, mean distances from bony landmarks to the knee joint line has a strong correlation with body stature, gender, or race [7]. As seen in this study, all distances had a wide SD, which decrease the reliability of using them as a guide for the knee joint line in a surgical setting. For example, the distance *D* in the current study had a comparable mean to other studies from difference races (Table 4) but had wide range from 3 mm to 30.7 mm.

**Table 4:** Investigated distances to the knee joint line in the current study and other studies

Parameters	Current study (Thai)	Fan et al. (Chinese)	Servien et al. (Caucasian)
Distance *A*	27.1 ± 2.7 (19.3–34)	26.04 ± 2.83	28.27 ± 2.59 (23–34.59)
Distance *B*	21.7 ± 2.5 (16.1–29.2)	23.62 ± 2.70	23.00 ± 2.29 (16.97–28.26)
Distance *C*	12.6 ± 3 (4.5–21.6)	18.48.8 ± 3.89	14.11 ± 3.04 (4.51–22.13)
Distance *D*	21.3 ± 3.6 (3–30.7)	23.45 ± 3.74	21.89 ± 3.09 (10.61–32.09)
Distance *E*	7.6 ± 4.8 (−3 to 19.9)	13.04 ± 5.16	NA
FW	76.7 ± 3.99 (57.9–93.8)	79.61 ± 6.6	81.72 ± 6.93 (66.73–99.37)

Mean ± SD (range)

All measurements were performed in millimeter

To overcome the weakness of distances from landmarks to the joint line, Servien et al. [8] suggested converting these measured distances into a ratio of the femoral transepicondylar width (epicondylar ratio), which will be more reliable and have less variation. We compared epicondylar ratios in the current study with other studies (Table 5) and found that our data came in line with those studies. However, the standard deviation and range of *C*/FW (0.05/0.05–0.3), *D*/FW (0.04/0.04–0.3), and *E*/FW (0.04/0.05–0.27) were still wider than other parameters. Many studies supported our results that *C*/FW and *E*/FW had a high standard variation, which is independent of patient size, and concluded that fibular head and inferior pole patellar are not reliable landmarks to guide joint line level in revision TKA [8, 9]. Furthermore, the fibular head is difficult to access during surgery due to its location and thick soft tissue coverage. The ratio of *D*/FW in many studies seems to be reliable for use as the tibial side landmark [8–10]. In contrast, our results demonstrated that *D*/FW had a high SD, wide range, and a significant difference between males and females (Table 3). The differences between our outcomes and previous research may be explained by variation in the ethnicity of participants, participant selection, measurement methodology, or observer differences.

**Table 5:** Epicondylar ratio from the current study and other studies

Parameters	Current study (Thai)	Fan et al. (Chinese)	Servien et al. (Caucasian)
*A*/FW	0.35 ± 0.02 (0.29–0.44)	0.327 ± 0.024	0.34 ± 0.02 (0.28–0.42)
*B*/FW	0.29 ± 0.02 (0.22–0.33)	0.297 ± 0.024	0.28 ± 0.02 (0.23–0.34)
*C*/FW	0.16 ± 0.05 (0.05–0.30)	0.232 ± 0.046	0.17 ± 0.04 (0.05–0.29)
*D*/FW	0.28 ± 0.03 (0.04–0.37)	0.295 ± 0.040	0.27 ± 0.03 (0.14–0.36)
*E*/FW	0.09 ± 0.04 (0.05–0.27)	0.165 ± 0.066	NA

Mean ± SD (range)

These ratios can be effectively used in real operative settings. Intraoperatively, the surgeon can use a ruler or vernier calipers to measure FW from the patient’s distal femur, then multiply by the epicondylar ratio of the relevant bony landmark calculated in this study (Table 3) to get an accurate joint line distance from that landmark. For example, if a surgeon performing revision TKA in a male patient would like to know the joint line distance from the medial epicondyle, they can use a ruler to intraoperatively measure the FW between the patient’s epicondyles then use the *A*/FW ratio calculated in our study (0.35 in Table 3) multiplied by the FW of the patient, which gives distance *A* for the patient. Using these epicondylar ratios in clinical practice can account for variation among individuals.

A limitation of the current study was the fact that measurements were performed based on MRI data. In MRI imaging, each landmark may not be located in the same plane of the most distal or posterior point of the joint line, which can make MRI measurements different from measurements made in 3D samples, such as 3D imaging, cadavers, or intraoperative measurements. In addition, the precise bony landmarks can be difficult to find intraoperatively in some situations, which may result in less accuracy of knee joint line level restoration. Instead of using these landmarks solely during the intraoperative period, we encourage surgeons to use them from the preoperative planning period to get tentative information about the level of the knee joint line and the position of prostheses, which can then be reconfirmed with intraoperative measurements. Furthermore, the surgeon should consider using more than one bony landmark as a guide for the joint line level in revision knee surgery.

## Conclusion

The present MRI study investigated the distances between the bony landmarks and the knee joint line, including the medial epicondyle, the lateral epicondyle, the fibular head, the tibial tubercle, and the inferior patellar pole in Thai knees. There were wide variations in standard deviation (SD) of all measured distances, which might not be suitable as references for the knee joint line identification in difficult or revision TKA. In contrast, the ratios of investigated distances related to the FW (epicondylar ratio) provided less variable means and SD than those of distance measurements. Among all the epicondylar ratios, the ratio between lateral epicondyle to joint line distance (distance *B*)/FW demonstrated the narrowest range of mean and SD values; therefore, this could be the most reliable landmark for intraoperative knee joint line verification by multiplying the FW of the patient by 0.29 to get distance *B* for that patient.

## Data Availability

The dataset during and/or analyzed during the current study are available from the corresponding author on reasonable request.

## Abbreviations

Distance *A*: The distance from medial femoral epicondyle to knee joint line
Distance *B*: The distance from lateral femoral epicondyle to knee joint line
Distance *C*: The distance from tip of fibular head to knee joint line
Distance *D*: The distance from tibial tubercle to knee joint line
Distance *E*: The distance from lower pole patellar to knee joint line
FW: Femoral transepicondylar width
LE: Lateral femoral epicondyle
ME: Medial femoral epicondyle
MRI: Magnetic resonance imaging
SD: Standard deviation
TKA: Total knee arthroplasty
